# Long noncoding RNAs are associated with metabolic and cellular processes in the jejunum mucosa of pre-weaning calves in response to different diets

**DOI:** 10.18632/oncotarget.24898

**Published:** 2018-04-20

**Authors:** Rosemarie Weikard, Frieder Hadlich, Harald M. Hammon, Doerte Frieten, Caroline Gerbert, Christian Koch, Georg Dusel, Christa Kuehn

**Affiliations:** ^1^ Leibniz Institute for Farm Animal Biology (FBN), Dummerstorf, Germany; ^2^ University of Applied Sciences, Bingen, Germany; ^3^ Educational and Research Centre for Animal Husbandry, Hofgut Neumühle, Münchweiler, Germany; ^4^ Faculty of Agricultural and Environmental Sciences, University Rostock, Rostock, Germany

**Keywords:** long noncoding RNA, transcriptome, jejunum, pre-weaning calf, nutrition

## Abstract

Long noncoding RNAs (lncRNAs) emerged as important regulatory component of mechanisms involved in gene expression, chromatin modification and epigenetic processes, but they are rarely annotated in the bovine genome.

Our study monitored the jejunum transcriptome of German Holstein calves fed two different milk diets using transcriptome sequencing (RNA-seq). To identify potential lncRNAs within the pool of unknown transcripts, four bioinformatic lncRNA prediction tools were applied. The intersection of the alignment-free lncRNA prediction tools (CNCI, PLEK and FEELnc) predicted 1,812 lncRNA transcripts concordantly comprising a catalogue of 1,042 putative lncRNA loci expressed in the calves’ intestinal mucosa.

Nine lncRNA loci were differentially expressed (DE lncRNAs) between both calf groups. To elucidate their biological function, we applied a systems biology approach that combines weighted gene co-expression network analysis with functional enrichment and biological pathway analysis. Four DE lncRNAs were found to be strongly correlated with a gene network module (GNM) enriched for genes from canonical pathways of remodeling of epithelial adherens junction, tight junction and integrin signaling. Another DE lncRNA was strongly correlated with a GNM enriched for genes associated with energy metabolism and maintaining of cellular homeostasis with a focus on mitochondrial processes.

Our data suggest that these DE lncRNAs may play potential regulatory roles in modulating biological processes associated with energy metabolism pathways and cellular signaling processes affecting the barrier function of intestinal epithelial cells of calves in response to different feeding regimens in the pre-weaning period.

## INTRODUCTION

Long noncoding RNAs (lncRNAs) are loci located in genomic regions, which are antisense, intronic, intergenic or overlapping with regard to protein-coding loci. They emerged as important components of mechanisms involved in various biological processes modulating developmental, metabolic and immunological changes. LncRNAs turned out to be functionally associated with specific developmental stages in cells and tissues, the pathogenesis of various diseases (e.g., tumor growth, mental and neurogenerative disorders, cardiovascular pathologies), the susceptibility to infection and other environmental challenges and also with metabolic disorders (e.g., obesity, diabetes) [1–13]. In these remarkably distinct biological processes, dysfunctions and conditions, lncRNAs were found to be involved in a broad range of mechanisms regulating gene expression, genomic imprinting, chromatin modification and epigenetic processes [14–16]. In spite of their potential functional relevance, even in human and mouse, most known lncRNAs are not functionally characterized.

In contrast to human and laboratory model species, lncRNAs are incompletely annotated in the reference genomes of domesticated and farm animals [17]. Transcriptome analyses by RNA sequencing (RNA-seq) have demonstrated to be useful for the identification of complex transcript catalogues of specific cells and tissues, including lncRNAs. The holistic RNA-seq approach allows us to discover previously undetected transcripts and to unravel novel regulatory mechanisms at the transcriptional level. There are only a few transcriptome studies with a focus on the identification and characterization of lncRNAs in specific bovine tissues using RNA-seq [18–23], and the most comprehensive catalogue of lncRNAs across tissues in cattle is based on data from a single animal [24]. Thus, identification and functional characterization of lncRNA atlases fits the aim of the international global network for Functional Annotation of Animal Genomes (FAANG), which is to identify and functionally annotate novel regulatory elements in domesticated animal genomes with a focus on biologically important representative tissues to generate a link between genome and phenome [25, 26].

In this context, the aim of our study was focused on the identification of lncRNAs and the elucidation of their potential regulatory function in intestinal tissue of calves in response to nutritional intervention by following-up a previous whole transcriptome study. In that study we had used RNA-seq to examine the transcriptional changes of protein-coding genes in response to different milk feeding regimens in calves during the pre-weaning period [27]. Our present study followed up previous reports (e.g., [1, 4, 6, 9, 12]) suggesting that lncRNAs are involved as regulatory integral component in the modulation of immunological and metabolic processes as well as in developmental and cellular proliferation. Thus, we hypothesized that lncRNAs may possibly play a regulatory role in mediating the effects of divergent early life milk supply in the gastrointestinal system of calves in the pre-weaning period. Our previous RNA-seq analysis had indicated that in the jejunal mucosa of calves the most divergent transcriptional response to restricted compared to *ad libitum* milk access was elicited by genes acting in the immune system. In contrast, the response of different milk supply was less pronounced on the metabolic system level compared to the immune system. Pursuing our hypothesis on lncRNAs, this follow-up analysis of the RNA-seq dataset had a focus on the identification of lncRNAs present in the jejunum mucosa of pre-weaning calves. Furthermore, it aimed to elucidate if lncRNAs may play a functional role in the modulation of gene expression patterns caused by restricted milk access of calves at this early ontogenetic stage.

## RESULTS AND DISCUSSION

### Feed intake and growth performance

Feed intake and growth curves of the two differentially fed calf groups were described in detail in our previous reports [27, 28]. In brief, higher milk intake (transition milk and milk replacer) was accompanied by a faster increase of body weight in AL compared to RES calves. RES calves had a higher concentrate intake than AL calves at the end of the experiment. However, total dry matter intake (sum of milk, milk replacer and concentrate intake) was not different between AL and RES calves during the whole experimental period.

### Transcriptome sequencing

Statistics of whole transcriptome sequencing of jejunum mucosa samples were presented in detail in our previous report [27]. Essentially, a total of 6.8 billion quality-filtered reads were available for subsequent guided alignment to the *Bos taurus* genome assembly UMD 3.1.1. A total of 88.6% of reads were mapped uniquely to the bovine reference genome. Finally, the annotation-guided transcript assembly revealed 69,429 transcripts (corresponding to 25,954 gene loci), which were expressed in at least one sample across calf groups with a minimum of 10 reads. Out of them, 14,689 transcripts (corresponding to 11,413 gene loci) were not annotated in the *Bos taurus* reference genome assembly. These unknown transcripts were assigned to class code “u” according to the Cufflinks pipeline [29]. The majority of them were monoexonic transcripts, 4,782 transcripts (2,117 loci) consisted of more than one exon.

### Analysis of unannotated transcripts and identification of lncRNAs

**Figure 1:**
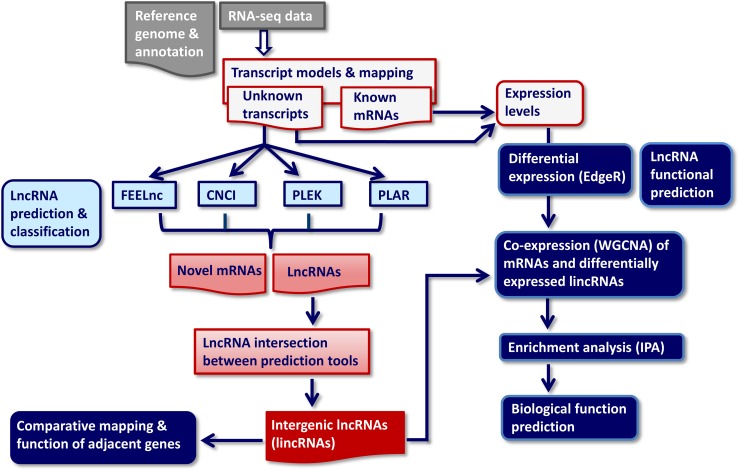
RNA-seq-based data analysis pipeline for identification, classification and biological function annotation of lncRNAs CNCI, PLAR, PLEK, FEELnc: Bioinformatic prediction tools. WGCNA: Weighted gene co-expression network analysis, IPA: Ingenuity enrichment and biological pathway analysis of genes in GNM significantly co-expressed with differentially expressed lncRNAs.

The transcripts not previously annotated in the bovine transcriptome were subjected to a RNA classification pipeline applying four independent bioinformatic tools in order to identify putative lncRNAs (Figure 1). The selected bioinformatic lncRNA prediction methods PLAR, PLEK, CNCI and FEELnc, are based on different intrinsic sequence-related features (composition, structural properties and motifs) and divergent filtering steps as has been reviewed recently [17]. They are mainly divided into alignment-free (CNCI, PLEK, and FEELnc) or alignment-dependent (PLAR) algorithms. A very critical filtering parameter for all lncRNA prediction tools is, whether intergenic transcripts with single exon structure were retained in the input dataset. Whereas CNCI discards all intergenic singletons, PLEK keeps them in the dataset and PLAR allows retaining only those single-exon transcripts fulfilling more stringent criteria (exonic length >2,000 nt and an FPKM >5 in at least one sample). FEELnc offers three options: remove all monoexonic transcripts, include all of them or keep only antisense singletons. Because we used an RNA-seq stranded protocol, we applied the third option for the analysis of our dataset with FEELnc.

The comprehensive lncRNA prediction analysis revealed that the classification of unknown transcripts is dependent on the specific bioinformatic tool applied (Table 1). It is striking that PLEK classified nearly all transcripts included in the input dataset of unknown transcripts (98%) compared to the other three tools (21%, 32% and 33% for PLAR, FEELnc and CNCI), which is due to the fact that monoexonic transcripts were not filtered out when applying PLEK. Thus, PLEK predicted the highest number of lncRNA transcripts to be present in the dataset of unknown transcripts. It is further noticeable that FEELnc and CNCI classified a similar number of putative lncRNA and novel mRNA transcripts, whereas PLAR resulted in the lowest number transcripts classified from the dataset of unknown transcripts (Table 1). The particularly remarkable low number of putative novel mRNA transcripts identified by PLAR could be due to the very stringent filtering parameters applied in this prediction algorithm.

**Table 1:** Analysis and classification of unknown transcripts applying different bioinformatic algorithms

Bioinformatic tool	Total number of classified transcripts^1^	Predicted lncRNAs^2^	%^4^	Predicted novel mRNAs^3^	%^4^
**FEELnc**	4,680	3,494	75	1,186	25
**CNCI**	4,784	3,626	76	1,158	24
**PLAR**	3,168	2,575	81	593	19
PLEK^*^	14,328	10,449	73	3,879	27

^1^Number of all transcripts classified by the respective bioinformatic algorithm from the input dataset comprising 14,689 class code “u” transcripts based on the filters specific for each tool, ^2^Number of putative lncRNAs (no coding potential) predicted from the total number of class code “u” transcripts and classified by the respective bioinformatic tool, ^3^Number of mRNAs (coding potential) predicted from the total number of class code “u” transcripts and classified by the respective bioinformatic tool, ^4^Percentage of transcripts relative to the total number of lncRNA and mRNA transcripts, respectively, classified by the respective bioinformatic tool, ^*^Singletons included.

**Figure 2:**
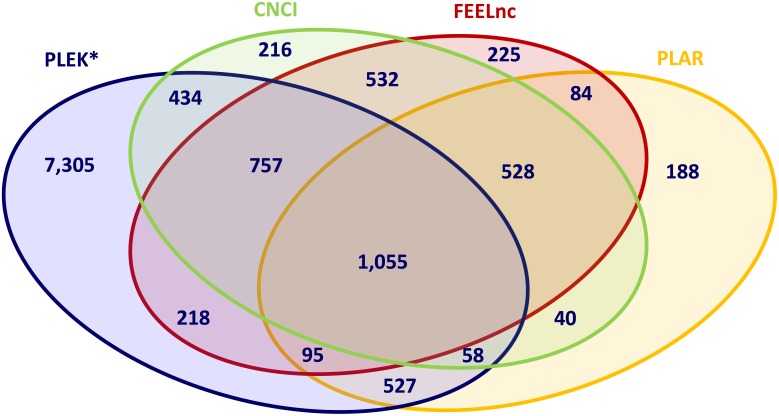
Intersection of predicted lncRNAs applying different bioinformatic prediction tools CNCI, PLAR, PLEK, FEELnc: Bioinformatic prediction tools.

After performing lncRNA prediction using these four algorithms separately, the intersection between the results from all four bioinformatic methods and all trio and pair combinations were determined in order to extract lncRNAs with high reliability and to evaluate the concordance between the lncRNA prediction tools applied. The intersection between all four tools extracted a total of 1,055 lncRNAs commonly predicted by all bioinformatic tools (Figure 2). This transcript set contains only intergenic lncRNAs (lincRNAs) with more than one exon because of the intrinsic filtering feature implemented in CNCI. The intersection between the trio combinations of lncRNA prediction tools revealed that the alignment-free pipelines, CNCI, FEELnc and PLEK, shared the highest number (1,812) of concordantly predicted of lncRNA transcripts (Supplementary Table 1). The pairwise intersection of bioinformatic pipeline showed the highest concordance between FEELnc and CNCI (2,872 lncRNA transcripts).

**Figure 3:**
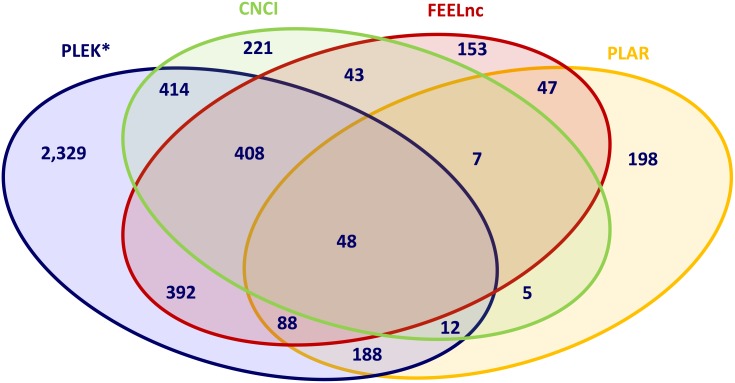
Intersection of predicted novel mRNAs applying different bioinformatic prediction tools CNCI, PLAR, PLEK, FEELnc: Bioinformatic prediction tools.

The intersection of novel mRNA transcripts predicted from the input dataset of unknown transcripts revealed that only 48 transcripts were concordantly predicted by all four prediction methods, and again the trio combination of the alignment-free pipelines, CNCI, FEELnc and PLEK, showed the highest concordance by predicting 457 novel mRNA transcripts corresponding to 204 novel mRNA loci (Figure 3, Supplementary Table 2). When using combinations of two prediction algorithms, the highest agreement in discovering novel potentially coding transcripts was displayed by the CNCI-PLEK pair.

Follow-up analyses of lncRNAs were performed using the intersection dataset from the alignment-free pipelines, CNCI, FEELnc and PLEK comprising 1,812 intergenic lncRNA transcripts corresponding to 1,042 putative lncRNA loci (lncRNA concordance dataset, Supplementary Table 1). The intersection dataset was used for sequence similarity searches in the *Bos taurus* genome NCBI assembly UMD3.1.1 and NONCODE 2016 databases in order to identify novel, unique bovine lncRNAs in our jejunal mucosa dataset. The results revealed that most of the lncRNA loci included in the lncRNA concordance dataset were novel. According to the selected identity thresholds and after manual inspection of identity hits we found that a total of 145 lncRNA loci detected in our study had substantial sequence similarity to 172 bovine lncRNA loci included in the NONCODE 2016 dataset. Out of these 145 confirmed lncRNA loci, 35 lncRNA loci from our dataset were nearly completely covered (>90%) by NONCODE lncRNAs (i.e., the total length of the NONCODE lncRNAs could be longer) (Supplementary Table 3). Finally, 897 out of putative 1,042 lncRNA loci from our lncRNA concordance dataset that were without substantial sequence similarity to sequences included in the NONCODE 2016 dataset, can be designated as novel.

### Differentially expressed lncRNAs

Transcriptome-wide differential expression analysis revealed that a total of 275 loci displayed differential expression between groups [27]. There were 55 loci in this dataset, which were not annotated in the bovine genome. Comparing the dataset of differentially expressed loci with our lncRNA concordance dataset, we retrieved nine lncRNA loci that were present in both datasets and met our stringency threshold to be reliably expressed in at least five samples (Table 2).

**Table 2:** lncRNA loci differentially expressed between calf groups fed different diets

Gene locus	AL (FPKM)	RES (FPKM)	Fold change (log2)	q-value	Chromosomal position (Mb)
XLOC_003822	1.14	0.31	−1.85	0.0664	11:105,094032-105,114596
XLOC_004079	5.52	1.38	−2.00	0.0085	11:25,480054-25,559973
XLOC_004376	9.26	0.57	−4.02	0.0085	11:77,900485-77,936781
XLOC_009175	16.96	5.06	−1.74	0.0085	16:46,076613-46,098065
XLOC_019876	1.79	0.21	−3.06	0.0085	23:40,796533-40,817043
XLOC_020293	0.62	0.08	−2.85	0.0522	24:7,206795-7,216215
XLOC_025957	43.33	14.50	−1.58	0.0988	3:104,408540-104,421736
XLOC_026410	23.28	6.61	−1.81	0.0085	4:77,906094-77,910010
XLOC_029089	9.77	3.56	−1.46	0.0085	5:104,398821-104,402665

FPKM (fragments per Kb per million reads).

These lncRNAs differentially expressed (DE lncRNAs) in the jejunal mucosa between both calf groups indicate a potential functional relevance for them in the modulation of regulatory processes in the calf intestine associated with adaptation to the different feeding regimen. All DE lncRNAs were downregulated in the RES calf group compared to the AL calves (Table 2).

Specific classification and mapping characteristics of these DE lncRNAs are summarized in Table 3. The DE lncRNA loci were generally represented by more than one transcript in our dataset comprising a length greater than 1 kb (except for XLOC_026410) and spanned one to five exons. They are localized between protein-coding genes and are classified as intergenic type (lincRNA) with a distance to the nearest annotated protein-coding gene varying between 106 and 87,187 bp.

**Table 3:** Characteristics of lncRNAloci differentially expressed between calf groups fed different diets

lncRNA locus	Nearest gene	Distance (bp)^1^	Location^1^	Class^1^	Direction^1^	Ntrans	Length (bp)	Nex
XLOC_003822	CACNA1B	39,145	Upstream	intergenic	sense	5	1,738-12,651	1-3
XLOC_004079	ZFP36L2	66,069	Down-stream	intergenic	sense	18	1,125-5,523	1-4
XLOC_004376	APOB	28,164	Upstream	intergenic	antisense	2	1,857-2,126	3
XLOC_009175	ERRFI1	26,160	Upstream	intergenic	sense	5	1,231-1,946	2-3
XLOC_019876	MYLIP	5,561	Upstream	intergenic	sense	3	2,714-2,906	2-3
XLOC_020293	SOCS6	87,187	Down-stream	intergenic	sense	3	1,012-1,523	1-3
XLOC_025957	RIMKLA	4,046	Upstream	intergenic	sense	13	740-6,380	1-5
XLOC_026410	POLM	499	Upstream	intergenic	sense	1	879	2
XLOC_029089	TNFRSF1A	106	Upstream	intergenic	antisense	4	2,784-2,936	3

^1^Classification of lncRNAs relative to the nearest gene was retrieved from FEELnc, Ntrans: number of transcripts per locus, Nex: number of exons.

Sequence similarity search of DE lncRNAs against the NONCODE 2016 database indicated that only XLOC_026410 is already deposited there. Screening the current bovine UMD 3.1.1 genome assembly at NCBI (annotation release 105, 10/2017) revealed that the DE lncRNAs (except for XLOC_019876 and XLOC_003822) displayed sequence similarity to noncoding RNA sequences predicted by automated computational analysis using the NCBI eukaryotic gene prediction tool Gnomon (Table 4).

**Table 4:** Sequence similarity of lncRNA loci differentially expressed between calf groups fed different diets

lncRNA locus	Similarity^1^ (Bos taurus)	Similarity^2^ (Other ruminants)	Locus^2^	Seq_ID^2^	Human synteny region^3^
XLOC_003822	NA	*Odocoileus virginianus texanus* *Ovis aries musimon*	LOC110123124 LOC105614597	XR_002309436 XR_001038849	NA
XLOC_004079	XR_809672 XR_809673	NA			HSA2, ZFP36L2, LINC02580, LINC01819
XLOC_004376	XR_236455 XR_809902	*Ovis aries* *Capra hircus*	LOC104989019 LOC105607652 LOC105607652 LOC106502644	XR_825310 XR_001028320 XR_001036966 XR_001296278	HSA2, APOB/TDRD15
XLOC_009175	XR_811953	*Bison bison* *Bos indicus*	LOC104995879 LOC109570778	XR_826114, XR_826113 XR_002182547	HSA1, ERRFI1 LOC107984914, LOC107984915
XLOC_019876	NA	NA			HSA6, MYLIP LINC02543, LOC105374949
XLOC_020293	XR_239769 XR_815095	*Bubalus bubalis*	LOC102410412	XR_327783	HSA18, SOCS6 LIVAR, LIN01909, LINC01910
XLOC_025957	XR_805801 XR_805800 XR_234114	*Bos mutus*	LOC106701547	XR_001351942	HSA1, RIMKLA/FOXJ3
XLOC_026410	XR_806237 NONBTAT030596	NA			HSA7, POLM/AEBP1
XLOC_029089	XR_139312 XR_234722	*Ovis aries musimon* *Odocoileus virginianus texanus*	LOC102340266 LOC102178050 LOC10560858 LOC110128121	XR_318473 XR_001918112 XR_001295742 XR_001030063, XR_001030062 XR_002311063	HSA12, TNFRS1A/SCNN1 LOC107984500

^1,2^Sequence similarity search against the NCBI and NONCODE databases, ^3^synteny search against the NCBI database, NA: no similarity or synteny.

Sequence similarity search of DE lncRNAs in the NCBI nucleotide database (nr, species other than human and mouse) discovered a robust evolutionary conservation (>90% identity) to lncRNAs from other ruminant species for all DE lncRNAs but not XLOC_019876, XLOC_004079 and XLOC_026410 (Table 4). Screening the current human genome assembly at NCBI (GRCh38.p7 primary assembly, annotation release 108, 10/2017) identified no similar human lncRNA sequences.

However, inspecting genomic regions adjacent to lncRNAs on human chromosomes that are syntenic to the targeted bovine chromosomal regions unveiled a similar structural architecture regarding the annotation of some DE lncRNAs (Table 4). XLOC_004079, XLOC_009175, XLOC_019876, XLOC_020293 and XLOC_029089 and corresponding human lncRNAs were found to be located in the neighbourhood of respective orthologous protein-coding genes. Generally, lncRNAs are known to have a low cross-species sequence conservation rate and lack common sequence features or structural motifs. However, the positional, orthologous conservation of lncRNAs and lincRNAs across vertebrates is reported in various species [30–32]. Human lncRNA LINC01910 is particularly interesting because it showed a three exon structure like XLOC_020293 and is located near SOCS6 on HSA18, a syntenic organization found for XLOC_020293 and SOCS6 on BTA24. In addition, LINC01910 was found to be specifically expressed in human small intestine [33] and showed a specific expression pattern during fetal development in this tissue [34].

To elucidate tissue specificity of DE lncRNAs discovered in our study we took advantage from cow RNA-seq data available at the *Bos taurus* UMD3.1.1 NCBI Genome Data Viewer, GCF000003055.6, (https://www.ncbi.nlm.nih.gov/genome/gdv/browser/?acc=GCF_000003055.6&context=genome). In addition, we searched for expression of DE lncRNAs in own unpublished RNA-seq datasets available for tissues from male and female animals originating from a Charolais x German Holstein cross. We found that all DE lncRNAs revealed expression in one of the other RNA-seq datasets and in more than one tissue (Supplementary Table 4). Hence, it can be excluded that they are specifically expressed only in calf jejunum.

### Functional analysis of differentially expressed lncRNAs

For inferring the regulatory functions of DE lncRNAs in the jejunal mucosa in response different nutrient regimes in calves we used a “guilt-by-association” approach which relies on similar co-expression profiles between lncRNAs and protein-coding genes of known function [35, 36]. Co-expression network analysis offers the possibility to simultaneously identify and investigate numerous genes displaying coordinated expression patterns at multiple experimental settings. To predict the functions of DE lncRNAs, we first constructed a co-expression network using the weighted correlation network analysis (WGCNA, see pipeline in Figure 1) and identified modules of co-expressed protein-coding genes (=gene network module (GNM), marked with different colors), which were subsequently correlated to DE lncRNA expression. The sample dendrogram resulting from WGCNA showed a clear clustering of calf groups based on expression levels of the nine DE lncRNAs (Supplementary Figure 1).

**Figure 4:**
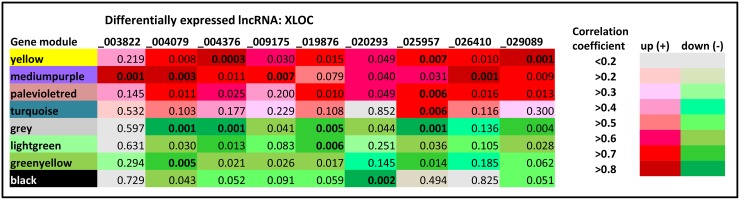
Weighted co-expression analysis of annotated and non-annotated loci with differently expressed lncRNAs Left column: Different colors represent different GNM identified by Weighted gene co-expression network analysis (WGCNA) established from pairwise correlations of gene expression patterns. Headline: Differentially expressed lncRNAs co-expressed with GNM. Significance of correlation is displayed by p-values, positive correlation is indicated in red, negative correlation is indicated in green, intensity of red/green colors corresponds to the magnitude of the correlation. Those GNM that are most highly co-expressed with at least one DE lncRNAs indicated by a correlation of r> |0.75| and p< 0.01 are displayed. Most significant correlations are given in bold.

The co-expression analysis revealed a total of 58 correlated GNM (Supplementary Figure 2), out of which 26 were significantly correlated (p< 0.05) with at least one of the lncRNAs included in the analysis. The GNM that are most highly co-expressed with at least one DE lncRNAs as indicated by a correlation of r> |0.75| and p< 0.01 are displayed in Figure 4. The DE lncRNA-gene module relationships showed that most of lncRNAs were correlated with several GNM, suggesting a co-regulation of the respective DE lncRNAs. The strongest DE lncRNA – gene module relationships were found for XLOC_004376. It was highly correlated to the gene modules “yellow” (r=0.883, p=0.0003) and “grey” (−0.861, p=0.0007) (Figure 4, Table 5). Both GNM displayed high correlations to several other DE lncRNAs. Out of them, XLOC_029089 revealed a strong correlation to gene module “yellow” (r=0.836, p=0.001), whereas XLOC_025957 and XLOC_004079 were strongly correlated with gene module “grey” (r=-0.839, p=0.001 and r=-0.838, p=0.001, respectively). However, there were also DE lncRNAs (XLOC_003822, XLOC_026410, XLOC_009175 and XLOC_0020293) that revealed a tight correlation with only a single GMN, indicating a specific transcriptional co-regulation. Whereas the first three DE lncRNAs were jointly correlated with the GNM “mediumpurple”, XLOC_0020293 was only significantly correlated with GNM “black” (r=-0.823, p=0.002). XLOC_0020293 seems to play a distinct regulatory role because in turn, GNM “black” revealed no strong correlation with any of the other DE lncRNAs.

**Table 5:** Gene modules and corresponding canonical pathways associated with DE lncRNAs

GNM	Gene number^1^	Co-expressed DE lncRNAs^2^	Correlation^2^	Top canonical pathways associated with gene modules	P-value
black	737	XLOC_020293	−0.823	Oxidative phosphorylation Mitochondrial dysfunction NRF2-mediated oxidative stress response TCA cycle II Glycolysis I	5.10E-46 8.56E-44 7.00E-05 1.88E-04 2.19E-04
yellow	967	XLOC_004376 XLOC_004079 XLOC_029089 XLOC_025957	0.883 0.751 0.836 0.761	Remodeling of epithelial adherens junctions Tight junction signaling Antiproliferative role of TOB in T cell signaling Integrin signaling	5.21E-08 2.37E-06 4.35$-06 5,84E-06
grey	186	XLOC_004376 XLOC_004079 XLOC_025957 XLOC_029089 XLOC_019876	−0.861 -0.838 -0.839 -0.789 -0.781	Bile acid biosynthesis, neutral pathway LPS/IL-1 mediated inhibition of RXR function Estrogen biosynthesis DNA methylation & transcriptional repression signalling	4.81E-04 1.55E-03 5.46E-03 9.56E-03
mediumpurple	48	XLOC_003822 XLOC_026410 XLOC_004079 XLOC_009175	0.844 0.848 0.805 0.758	Retinoic acid mediated apoptosis signaling Death receptor signaling	4.94E-03 1.07E-02
greenyellow	453	XLOC_004079	−0.772	Role of PKR in interferon induction & antiviral response PPAR signaling TNFR2 signaling TNFR1 signaling	1.05E-05 1.25E-05 1.76E-05 4.03E-05
paleviolet	26	XLOC_025957	0.770	S-methyl-5′-thioadenosine degradation II Glutamine biosynthesis I	6,06E-03 7.06E-03
turquoise	1,616	XLOC_025957	0.771	Estrogen receptor binding Assembly of RNA polymerase III complex Protein ubiquitination pathway Assembly of RNA polymerase II complex	7.23E-05 8.40E-05 1.45E-04 2.88E-04
lightgreen	72	XLOC_019876	−0.763	PI3K/AKT signalling	5.25E-03

^1^Number of genes (in the GNM) with known function, ^2^p < 0.01, r > ±0.75.

The “guilt-by-association principle” claims that genes sharing the same function or that are involved in the same regulatory pathway will tend to present similar expression profiles and hence, form clusters or modules in the network [36]. Thus, within the same module, genes of known function can be used to predict the function of co-expressed unknown genes [14, 35]. For functional annotation of lncRNAs expressed in the jejunum mucosa, co-expression analysis of DE lncRNAs and mRNAs (protein-coding genes) with known biological function was linked with enrichment and canonical pathway analysis of GNM, which were significantly correlated to DE lncRNAs using IPA (see pipeline in Figure 1).

**Figure 5:**
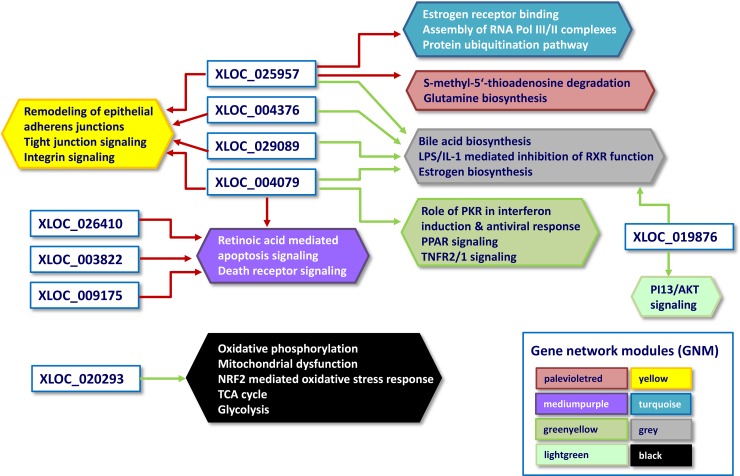
Network of differentially expressed lncRNA and their linked biological pathways predicted by biological pathways analysis Arrows: Positive correlation between gene network modules (GNM)/biological pathways and DE lncRNAs identified by Weighted gene co-expression network analysis (WGCNA) is indicated in red, negative correlation is indicated in green. Hexagons represent the canonical pathways of GNM identified by Ingenuity pathway analysis, their different colors are equivalent to those of the network modules correlated with DE lncRNAs.

The canonical pathways identified for the most tightly co-expressed DE lncRNA – GNM are summarized in Table 5; and Figure 5 illustrates the integrated network summary of most significantly co-expressed DE lncRNA- GMN pairs. The results obtained from the combined co-expression - biological pathway analysis (WGCNA-IPA) suggest that the DE lncRNAs might be involved in various different biological pathways modulated in the calves’ jejunal mucosa in response to different milk diets. Based on known functions of the co-expressed protein-coding genes, hypotheses can be generated for the functions and potential regulators of the DE lncRNA [14, 35]. Some of the most interesting DE lncRNAs – biological pathway relationships are elucidated and discussed in more detail.

**Figure 6:**
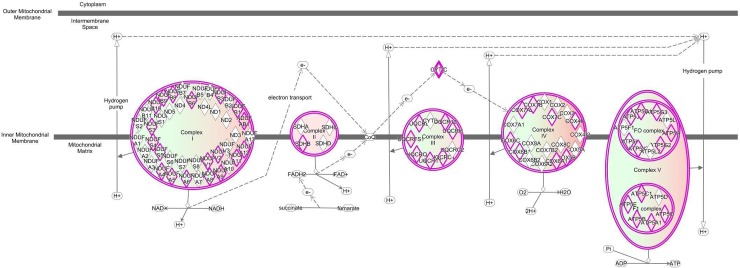
Ingenuity canonical pathway “Oxidative phosphorylation” is predicted to be modulated by DE lncRNA XLOC_02093

Most interesting out of the nine DE lncRNAs is XLOC_020293 because it was strongly correlated to a single GNM indicating a potential regulatory connection to metabolic pathways. This GNM “black” comprised genes, which were highly significantly enriched in the Ingenuity canonical pathways “Oxidative phosphorylation” and “Mitochondrial dysfunction” (Table 5, Figures 5 and 6). Other genes included in the GNM “black” were associated with canonical pathways “NRF2-mediated oxidative stress response”, “TCA cycle II” and “Glycolysis I” (Table 5, Figure 5), which are collectively implemented within cellular energy production. The strong negative correlation between the expression level of DE lncRNA XLOC_020293 and genes included in GNM “black” suggests a potential regulatory function of this DE lncRNA in metabolic processes related to energy metabolism and maintaining of cellular homeostasis with a specific focus on mitochondrial processes. This is particularly imperative for intestine tissue, because it has an intense metabolic rate and has high energy expenditure required for digestion and absorption processes [37]. The small intestine possesses adaptive capacity to adjust form and function in response to changes in digestive load [38]. As illustrated in Figure 6, all five complexes of the electronic transport chain (ETC) included in the pathways “Oxidative phosphorylation” and “Mitochondrial dysfunction”, are represented in the list of GNM “black” genes. We found that 53% of genes involved in the ETC were collectively modulated in response to the different diets. Most of the affected genes showed a tendency for higher expression levels in the RES calves than AL calves indicating an upregulated oxidative phosphorylation in RES calves. Similar transcriptional effects were observed for genes involved in the pathways “TCA cycle II” and “Glycolysis I” (Supplementary Figures 3, 4). In addition, as shown in Supplementary Figure 5, there are several other genes encoding mitochondrial proteins showing slightly higher but not significant gene expression levels in RES calves compared to AL calves (e.g*., FIS1, VDAC1, HSD17B10, AIFM1, PINK1, TXN2, PRX2, ACO2*). In summary, this indicates a coordinated modulation of biological processes in the intestinal mitochondria of RES calves, which might involve a regulatory role of the strongly co-expressed DE lncRNA XLOC_020293.

Analysis of potential upstream regulators for GNM “black” by IPA predicted RICTOR (RPTOR independent companion of mTOR complex 2) as potential candidate (z score: −5.93, p-value 4.66E-23). RICTOR and MTOR are components of a protein complex that integrates nutrient- and growth factor-derived signals to regulate cell growth [39]. Hence, it can be hypothesized that presumably DE lncRNA XLOC_020293 may interact with RICTOR to coordinately fine-tune biochemical processes regulating energy metabolism in response to different nutrient regimens in the calf groups.

There is evidence from studies in human and mouse that lncRNAs are implicated in differentiation and homeostasis of metabolic tissues in human and mouse [6, 40–45]. LncRNA-mediated regulation of metabolic processes related to glucose metabolism has been particularly discussed for aerobic glycolysis in cancer associated with the Warburg effect [46]. Goyal et al. [47] found an association of H19 lncRNA levels and impaired gluconeogenesis in diabetic mice. Lan et al. [48] reported that lnc-HC lncRNA plays a regulatory role in hepatocytic cholesterol metabolism by modulating the expression of *CYP7A1* and *ABCA1* genes implicated in cellular cholesterol excretion. In diabetic nephropatic mice and cell lines from different mouse tissues, Long et al. [49] discovered that the lncRNA TUG1 regulates *PPARGC1A* gene expression, the transcriptional coactivator that plays an integral role in maintaining energy homeostasis and mitochondrial biogenesis in response to a multitude of nutrient and hormonal signals. Several lncRNAs (ANRIL, AScmtRNA, H19, HOTAIR, LincRNA-p21, MALAT1, RMRP, SAMMSON, and VL30) have emerged as potent regulators of mitochondrial metabolism [50]. In contrast to human and mouse, studies in domesticated animals elucidating the biological function of lncRNAs associated with metabolic processes are limited. For instance in cattle, it has been reported that lncRNA ADNCR is known to act as competing endogenous RNA to sponge miRNA-204, thereby regulating the expression of the target gene *SIRT1*, which in turn results in inhibiting of bovine adipocyte differentiation [51]. In addition, several unknown lncRNAs potentially regulating fat metabolism in liver tissue of dairy cows were identified [23]. Our study is the first one with a specific focus of metabolism-associated lncRNAs in bovine gastrointestinal tissue. However, it has to be considered that the different effects between calf groups observed on transcriptional level of DE lncRNA XLOC_020293 and genes enriched in associated canonical pathways in response to different diets were recognized at the time point of sampling, two weeks after terminating the different milk feeding period. We cannot clearly distinguish, whether the effects on lncRNA expression level and the genes enriched in pathways linked to energy metabolism might be prolonged effects from the period of different diets on the intestinal epithelial cells or rather originate from different concentrate intake prior to sampling/slaughtering.

Table 5 and Figure 5 highlight DE lncRNAs XLOC_004376, XLOC_004079, XLOC_029089 and XLOC_025957, which were collectively positively correlated with the GNM “yellow”. This GNM comprises genes that were predominantly enriched in canonical pathways in “Remodeling of epithelial adherens junctions”, “Tight junction signaling”, “Antiproliferative role of TOB in T cell signaling” and “Integrin signaling”.

Adherens and tight junctions belong to the adhesive complexes connecting adjacent epithelial cells and intercellular space in the intestinal epithelium, which acts as a selectively permeable barrier. They prevent the passage of harmful intraluminal entities including foreign antigens, microorganisms and their toxins, and simultaneously permit the absorption of essential dietary nutrients, electrolytes and water from the intestinal lumen via the formation of complex protein-protein networks (reviewed by [52]). Rearranging adherens junctions is essential to drive epithelial remodeling during developmental and aging processes, when cells frequently change their shape and position relative to neighboring cells [53]. Defects in the adhesive characteristics of epithelial cells may affect assembly and disassembly of cell-cell adhesion and the ability of cells to regulate their adhesive interactions during tissue morphogenesis, repair and renewal; and these processes may play a key role during adaptive development of jejunal mucosa of calves in the postnatal and pre-weaning period and in response to different diets. In our study, remodeling activity of epithelial adherens junctions in intestinal mucosa tended to be different in both calf groups. Several genes acting in this pathway including *CDH1* and *CTNND1*, which play a critical role in formation of adherens and tight junctions and influence membrane surface stability [54], were sligthly lower expressed in RES compared to AL calves. Hence, the DE lncRNAs tightly co-expressed with the respective GMN could possibly be involved as regulatory component in this biological pathway “Remodeling of epithelial adherens junctions”.

Tight junction signaling is also involved in the modulation of connections between adjacent epithelial cells by participating in the regulation of cell proliferation and differentiation [55]. Tight junctions (TJ) are the most apical structure present in the junctional complex between the epithelial cells. This multifunctional complex was reported to contribute to the paracellular barrier and signal transduction in vertebrate epithelial and endothelial cells [56]. Claudins are the major structural and functional protein components of TJ directly regulating the paracellular passage of ions and solutes in-between cells in an epithelial layer (gate function) and determining the barrier properties [57, 58]. In our study a member of the claudin gene family, *CLDN4,* was significantly lower expressed in intestinal mucosa of RES calves than in AL calves. The corresponding protein CLDN4 is a tight junction-sealing claudin that was found to be expressed in differentiated luminal epithelial cells with a tight barrier [59]. In mouse intestine cells, Capaldo et al. [60] showed that cytokine-induced proinflammatory TJ remodeling is associated with increased CLDN4 dynamics at the TJ and contributes to epithelial barrier dysfunction by decreasing the assembly of CLDN4 into TJ. In studies that investigated barrier function in cows and calves, it was found that the expression of genes encoding TJ proteins, are affected by age and diet and that intestinal barrier function in calves is suggested to be compromised during the pre-weaning phase [61, 62]. Transcriptome analysis of small intestine of neonatal calves revealed significant temporal upregulation of *CLDN4* expression in the first week of life in the jejunum suggesting that barrier function changes immediately *post partum* at an early ontogenetic stage [63]. It has also been reported that gastrointestinal permeability decreases when calves age from 17 to 42 d, suggesting an improved barrier function [64].

The “Tight junction signaling” was also one of the most significantly enriched canonical pathways in our previous study [27], which had focused on containing differentially expressed coding genes in the jejuna of calves experienced different milk supply. Based on the knowledge from literature it can be suggested that downregulation of *CLDN4* in RES calves compared to AL calves in our study may support a tighter epithelial phenotype resulting in reduced paracellular permeability properties of the intestinal mucosa in AL calves compared with RES calves. The DE lncRNAs XLOC_004376, XLOC_004079, XLOC_029089 and XLOC_025957 were highly correlated with the GNM “yellow”, which was enriched for genes in the “Tight junction signaling” pathway. Thus, these DE lncRNAs might be involved in specific fine-tuning of TJ dynamics affecting mechanical strength of the intestine epithelium and might play a functional role in controlling the intestine permeability by altering the stability and translation of respective target mRNAs/genes in calves subjected to a different feeding regimen. Recent studies in human and mouse revealed that lncRNAs play a relevant role in controlling the intestinal epithelial barrier function. Overexpression of H19 lncRNA or silencing of SPRY4-IT1 lncRNA were reported to be accompanied by translational repression of genes encoding tight junction and adherens proteins, which led to dysfunction of epithelial barrier in intestinal cells [65, 66]. Post-transcriptional regulation of intestinal epithelial integrity by lncRNAs has been highlighted as functionally important for the maintenance of the gut epithelial integrity under changing environments requiring that epithelial cells rapidly elicit alterations in gene expression patterns to regulate their survival, adapt to stress and keep epithelial homeostasis [67].

Furthermore, we found that three of the DE lncRNAs (XLOC_004376, XLOC_004079, XLOC_029089 and XLOC_025957) that were strongly linked to epithelial adherens junction remodeling and TJ signaling pathways were also collectively negatively correlated with GNM “grey”. This module included genes that are involved in canonical pathways “Bile acid biosynthesis”, “LPS/IL-1 mediated inhibition of RXR function” and “Estrogen biosynthesis” (Table 5, Figure 5). Obviously, XLOC_004079 and XLOC_025957 seem to play a multi-functional role because they were also significantly correlated with two further GNM. XLOC_004079 was correlated with GNM predominantly enriched for genes associated with immune response signaling (interferon induction, antiviral response, TNFR signalling, GNM “greenyellow”) suggesting that XLOC_004079 might be involved in the regulation of the different transcriptional response observed in RES compared to AL calves in the jejunal mucosa of calves as reported in our previous study [27]. In addition, XLOC_004079 was strongly correlated with the GNM “mediumpurple” enriched for genes in the apoptosis signaling pathway. XLOC_025957 revealed significant correlation to GNM “turquoise” and “palevioletred”, indicating a function in “Estrogen receptor binding” and “Assembly of RNA polymerase II/III complexes” as well as “Protein ubiquitination pathway” and “S-methyl-5′-thioadenosine degradation II”. In contrast to the multiply interrelated XLOC_004079 and XLOC_025957, DE lncRNAs XLOC_026410, XLOC_003822 and XLOC_009175 revealed strong correlations with only a single GNM analogous to DE lncRNA XLOC_02093 (Table 5, Figure 5). The first three DE lncRNAs may exert a joint function together with XLOC_004079 in apoptosis signaling pathways (GNM “mediumpurple”) indicating the relevance of a meticulous fine-tuning of the related multifaceted biological processes.

## CONCLUSIONS

Altogether, our study provides the first catalogue of 1,042 potential lncRNA loci expressed in the jejunal mucosa of calves. Moreover, we identified nine lncRNAs that displayed different expression pattern in pre-weaning calves in response to different milk supply, and we predicted potential biological roles for DE lncRNAs in cellular signaling and metabolic processes associated with the different nutritional challenge of the calves. DE lncRNAs were predicted to be most likely linked to pathways essential for energy metabolism in the intestinal epithelium and to be associated with signaling pathways focusing on barrier function of intestinal epithelial cells. The DE lncRNAs correlated with GNM enriched with genes associated with these cellular and metabolic processes may represent specific biological transcriptional signatures in the jejuna of pre-weaning calves in response to different diets.

The results of our study will provide a piece of evidence supporting the FAANG initiative to accelerate the structural and functional annotation of noncoding regulatory elements in the bovine genome and deciphering genome to phenome relationships. Given the positional, orthologous conservation of lncRNAs across vertebrates, further investigation of the reproducibility and function of candidate lncRNAs can benefit from the increase in available RNA-seq data and comprehensive gene expression atlas datasets for cattle and other ruminant and livestock species (e.g., [68, 69]). Comparative model transcriptome resources across livestock species might also be valuable to better understand the function and regulation of orthologous human genes. However, additional validation of lncRNA roles in response to different feeding regimen has to be performed in future studies. Interrogating identified gene networks or pathways of interacting genes in more detail will be required to find the central players and identifying the interaction partners of prominent lncRNAs on genome, transcriptome and proteome level in the respective tissue and under different environmental conditions. Knowledge gleaned from this comprehensive analysis will be helpful to better understand the integral balance between the digestive system and nutrient digestion in response to dietary modulation of ruminants at an early stage of life.

## MATERIALS AND METHODS

### Animals, experimental design and sampling

The experimental design of the study has already been described in detail earlier [27, 28]. The animal experiment was conducted at the Educational and Research Centre for Animal Husbandry, Hofgut Neumuehle, Germany. In brief, twelve male German Holstein calves were reared from birth until day 80 (80.4 ± 1) of age. After initial colostrum feeding, the calves were divided into two feeding groups (6 animals each), which both were fed acidified transition milk (2 mL acidifier/L milk, H. W. Schaumann GmbH, Pinneberg, Germany) for three days. Starting from day four, one calf group (RES) had restricted access to milk replacer diet (6 L/d, milk replacer: 125 g powder per L; Trouw Nutrition Deutschland GmbH, Burgheim, Germany), whereas the second calf group (AL) received milk replacer *ad libitum* (max. 25 L/d) for the first eight weeks of age. Water was freely available; hay and concentrate (Raiffeisen, Köln, Germany) were offered *ad libitum* to all calves starting from 10 (±3) days of age. The amount of milk replacer was reduced linearly during week 9 to 10 in both groups and milk was fed in amounts of 2 L/d until the end of the experiment. The ingredients and chemical composition of milk replacer and concentrate and further experimental details were provided in our previous report [27]. Milk, milk replacer, and concentrate intake was documented daily from first to 11th week of age, and body weight was recorded weekly until the end of the experiment as recently described [27, 28]. At slaughtering (day 80), jejunum mucosa samples were collected by scraping the mucosa from the submucosa with a slide and immediately snap-frozen in liquid nitrogen.

### Transcriptome sequencing, transcript assembly and analysis of differential expression

Total RNA isolation, quality control of RNA and preparation of indexed, stranded sequencing libraries (polyA selection, TruSeq Stranded mRNA library preparation kit, Illumina) is described in more detail in our previous report [27]. In brief, the libraries were sequenced using a paired-end protocol (2 × 80 bp) on an Illumina HiSeq2500 sequencer platform. After demultiplexing, reads were trimmed for quality and adapter sequences with Cutadapt [70] and in-house linux tools. The reads passing quality control were subjected to further bioinformatics analyses. As described in our previous report [27], read alignment, transcript assembly and differential expression analysis essentially followed the pipeline published by Trapnell and co-workers [71]. Reads of all samples were aligned against the bovine reference genome assembly UMD3.1 (ftp://ftp.ensembl.org/../../pub/release-83/fasta/bos_taurus/dna/) by the Bowtie/Tophat2 pipeline using a guided annotation approach based on the Ensembl annotation 83 (ftp://ftp.ensembl.org/../../pub/release-83/gtf/bos_taurus/) as starting point. The aligned reads were assembled into contigs by Cufflinks2 and the resulting gtf files from all samples and the Ensembl gtf file were merged by Cuffmerge to create the final gtf transcript annotation file, which served for transcript quantification via Cuffdiff2. The guided assembly strategy was selected because of the incomplete *Bos taurus* genome annotation. In our previous study [27], cluster analysis, multidimensional scaling plot and inspection of individual transcripts demonstrated that one sample from the AL calf group was an outlier compared to the other samples. Therefore, the respective sample was removed from further analyses. Cuffdiff2 (pooled dispersion modelling) was applied to test for differential expression between both calf groups. Based on raw read counts as determined by Cuffdiff2, differences in transcript abundance between the two calf groups were also calculated using edgeR [72] as a second method to validate differentially expressed transcripts [73]. Differential expression analysis results were corrected for multiple testing [74] and considered as significant at q <0.1.

### Analysis and classification of unknown transcripts

The RNA-seq data subset representing those transcripts not previously annotated in the bovine transcriptome (class code “u” according to the Cufflinks package, [29]) served as input for the analysis and classification of unknown transcripts and the identification of putative lncRNAs.

To predict lncRNAs from whole transcriptome datasets, numerous computational bioinformatic algorithms and pipelines have been developed [17]. In our study, four different, independent bioinformatic lncRNA prediction tools were applied in parallel: three alignment-free algorithms, CNCI [**C**oding-**N**on-**C**oding **I**ndex, [75], https://github.com/www-bioinfo-org/CNCI)], PLEK [(**P**redictor of **l**ong non-coding RNAs and m**e**ssenger RNAs based on **k**-mer scheme, [76], https://sourceforge.net/projects/plek/files/)] and FEELnc [(**F**l**E**xible **E**xtraction of **l**ong **N**on**C**oding RNAs, https://github.com/tderrien/FEELnc, [77]], and the alignment-dependent algorithm PLAR [(**P**ipeline for **L**ncRNA **A**nnotation from **RNA-seq** data [78], http://www.weizmann.ac.il/Biological_Regulation/IgorUlitsky/pipeline-lncrna-annotation-rna-seq-data-plar)]. The selected bioinformatic lncRNA prediction tools extract different sequence-based features and attributes and apply specific filtering steps. For classification of unannotated transcripts applying the four lncRNA prediction tools, we used default parameters as implemented in the specific algorithms. For lncRNA prediction with CNCI we applied the human/vertebrate training dataset as recommended. Only intergenic transcripts with at least two exons were kept in the classification dataset after filtering. For our lncRNA analysis with PLEK we developed a bovine-specific model as suggested by the authors of PLEK. This model was calculated based on *Bos taurus* lncRNA sequences extracted from the NONCODE 2016 database (http://www.noncode.org/) and *Bos taurus* protein-coding mRNA sequences extracted from the NCBI *Bos taurus* UMD3.1.1 genome assembly (ftp://ftp.ncbi.nlm.nih.gov/genomes/Bos_taurus/) as input data). As implemented in PLEK, monoexonic transcripts were not excluded from the analysis. For the lncRNA analysis and classification with FEELnc, we also applied a bovine-specific training dataset based on *Bos taurus* lncRNA sequences from the NONCODE 2016 database and *Bos taurus* mRNA sequences (protein-coding biotype) extracted from the NCBI *Bos taurus* UMD3.1.1 genome assembly. Monoexonic transcripts (default parameter) were generally filtered out, except for those transcribed in antisense direction. The lncRNA analysis with PLAR required downloading respective annotation files for *Bos taurus* Refseq genes, other Refseq genes, *Bos taurus* RepeatMasker and chromosome sizes from the UCSC Genome Browser suite (http://genome.ucsc.edu/). Files required regarding features and structures of *Bos taurus* transcripts were downloaded from the Ensembl Biomart interface (http://www.ensembl.org/biomart/martview/4af292cc38e3f22b21a6cec10ea38471) as recommended.

After performing lncRNA prediction using these four algorithms separately, the intersection between all four bioinformatic tools, and combinations consisting of two and three of them were determined.

### Identification of novel yet unreported lncRNAs

Sequence similarity searches with putative lncRNA transcript sequences retrieved from the intersection of the alignment-free lncRNA prediction tools, CNCI, PLEK, and FEELnc, were performed using the BLASTN algorithm against *Bos taurus* lncRNA sequences deposited in the NONCODE 2016 database (http://www.noncode.org/). The E-value cut-off for a BLAST top hit was considered significant at less than 10E-100, and a stringent threshold for sequence identity was defined to be ≥98% in a region covering ≥100 nucleotides. In a second step, these results were manually curated by localizing those NONCODE lncRNA sequences with significant similarity to positions of lncRNAs from our study in the NCBI *Bos taurus* UMD3.1.1 reference genome (annotation release 105) using MEGABLAST (https://blast.ncbi.nlm.nih.gov/Blast.cgi) with default parameters. Sequence or locus identity was accepted, if mapping coordinates of the lncRNA sequence from our study and the respective bovine lncRNA locus deposited in the NONCODE 2016 database were concordant. Finally, we extracted those lncRNA sequences from our analyses, which were nearly completely covered by the respective NONCODE lncRNA or vice versa. Highly similar NONCODE lncRNAs had to have nearly full length coverage (>90%) by jejunal lncRNA transcripts from our dataset (i.e., total length of the jejunal lncRNAs could be longer), or lncRNA transcript sequences from our dataset were nearly completely covered (>90%) by NONCODE lncRNAs (i.e., the total length of the NONCODE lncRNAs could be longer) were considered to have full identity.

### Co-expression analysis

Weighted gene co-expression network analysis (WGCNA [79]) implemented in the R package WGCNA [80], version 1.61, has been performed to construct a weighted correlation network with protein-coding genes expressed in the calf jejunum transcriptome. Therefore, all gene loci (comprising annotated and unannotated loci) with average normalized FPKM (Fragments per kilo base transcript per million reads) values >0.1 in at least five samples were included in the analysis. An adjacency matrix of pairwise correlations between expression levels of all pairs of genes across all samples was generated, reporting the connection strength between gene pairs. We selected a soft thresholding power β for constructing weighted gene network as calculated by the picSoftThreshold function. The respective scale-free topology index reached saturation at a value of 20. Network modules, designated with different colors, were established by hierarchical clustering. For the module generating function, blockwiseModules, we selected a minimum module size of 30, and a threshold for merging modules of 0.25 while keeping all other parameters at default.

The hypothesis is that genes, which are highly interconnected within a network eigengene module, are generally involved in the same or linked biological pathways. Finally, these gene network modules (GNM) were related to lncRNAs differentially expressed between RES and AL calf groups by determining the correlation between the module eigengene values, which are the first principal components of the gene expression data within the modules, with lncRNA expression levels (co-expression correlation). Differentially expressed lncRNAs were considered to be significantly correlated with a GNM at p <0.05.

### Pathway and network analysis

To identify putative lncRNA-related biological pathways and to predict the functional roles of differentially expressed lncRNAs, we performed enrichment, pathway and network analyses using the Ingenuity analysis package (IPA, https://www.qiagenbioinformatics.com/products/ingenuity-pathway-analysis/). Therefore, genes from significantly correlated GNM identified previously by WGCNA were used as input for the IPA analysis. All annotated protein-coding genes of a GNM were included in the analysis, unannotated loci were discarded. We manually edited gene names of those transcripts only annotated with Ensembl annotation ID numbers according to the Ensembl *Bos taurus* annotation release 90 and NCBI *Bos taurus* annotation release 105.

### Ethics approval and consent to participate

The study was approved by the local department for animal welfare affairs (23 177-07/G 13-20-069, Landesuntersuchungsamt, Koblenz, Germany) in conformity with the German Animal Welfare Act.

## SUPPLEMENTARY MATERIALS FIGURES AND TABLES










