# Supplementary material for: Long noncoding RNAs are associated with metabolic and cellular processes in the jejunum mucosa of pre-weaning calves in response to different diets
Source: Oncotarget. 2018 Apr 20;9(30):21052–69. doi: 10.18632/oncotarget.24898 (PMC5940403; doi:10.18632/oncotarget.24898)
Supplement: Supplementary file 1 [file oncotarget-09-21052-s001.pdf]

# Long noncoding RNAs are associated with metabolic and cellular processes in the jejunum mucosa of pre-weaning calves in response to different diets

## SUPPLEMENTARY MATERIALS

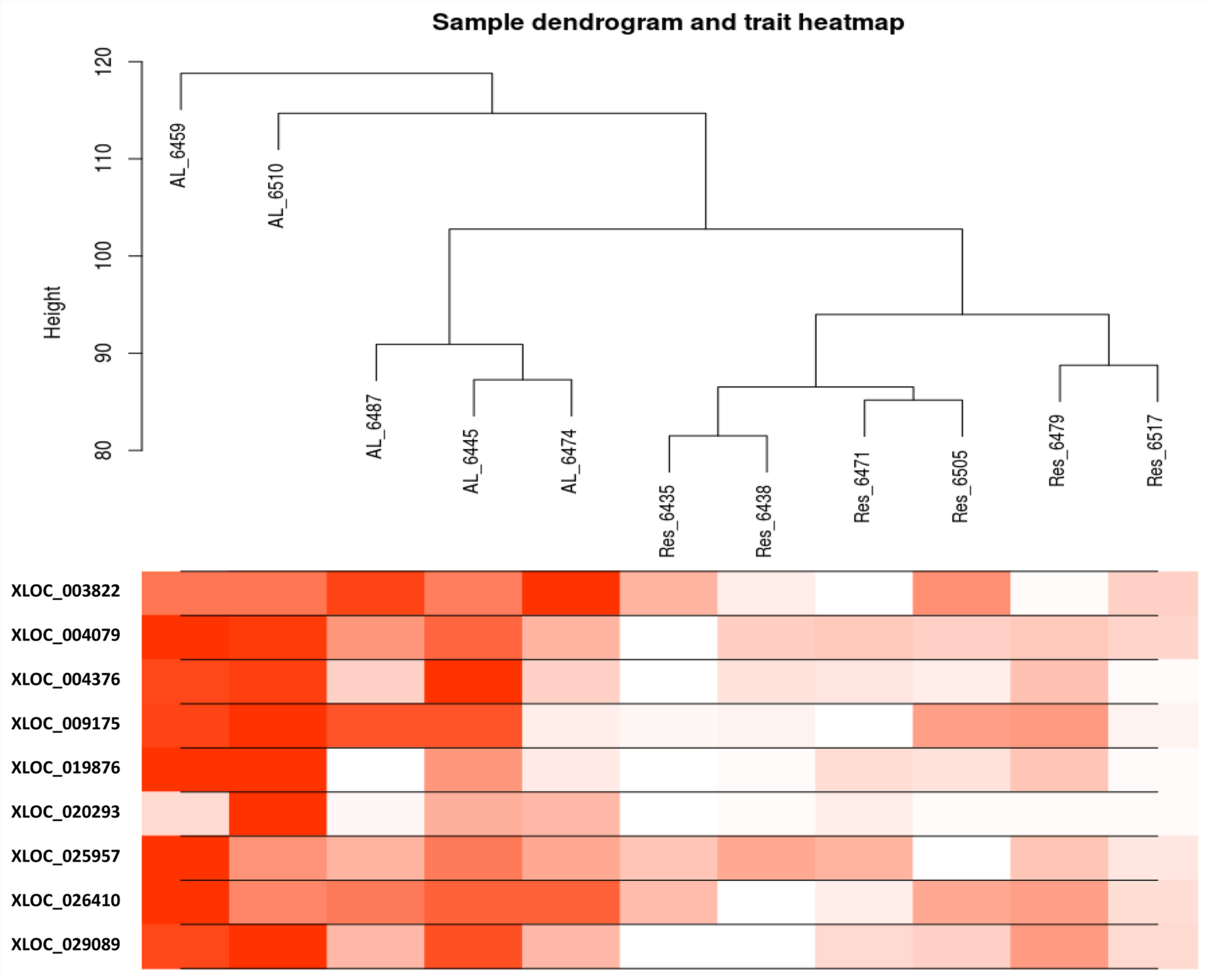


**Supplementary Figure 1: Sample dendrogram based on cluster analysis of RNA-seq expression levels using Weighted gene co-expression network analysis (WGCNA).** Red color indicates expression levels of the nine DElncRNAs in the samples; the stronger the color, the higher the expression is.

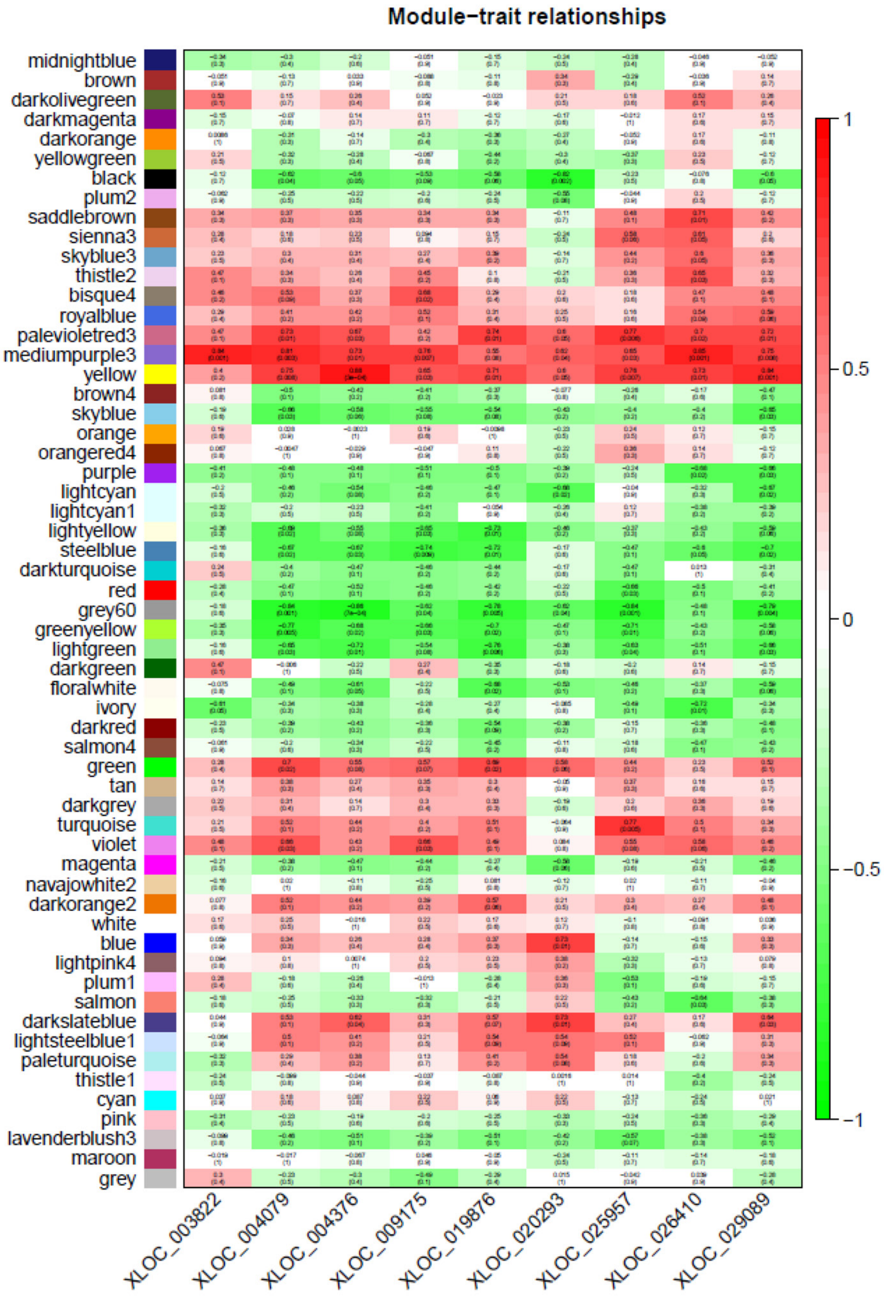


**Supplementary Figure 2: Weighted co-expression analysis of annotated and non-annotated loci with differently expressed lncRNAs.** Left column: Different colors represent different GNM identified by Weighted gene co-expression network analysis (WGCNA) established from pairwise correlations of gene expression patterns. Positive correlation is indicated in red, negative correlation is indicated in green, intensity of red/green color corresponds to correlation magnitude. Numbers in the colored cells indicate the correlation coefficient r and the level of significance (p value, the stronger the color, the more significant the correlation).

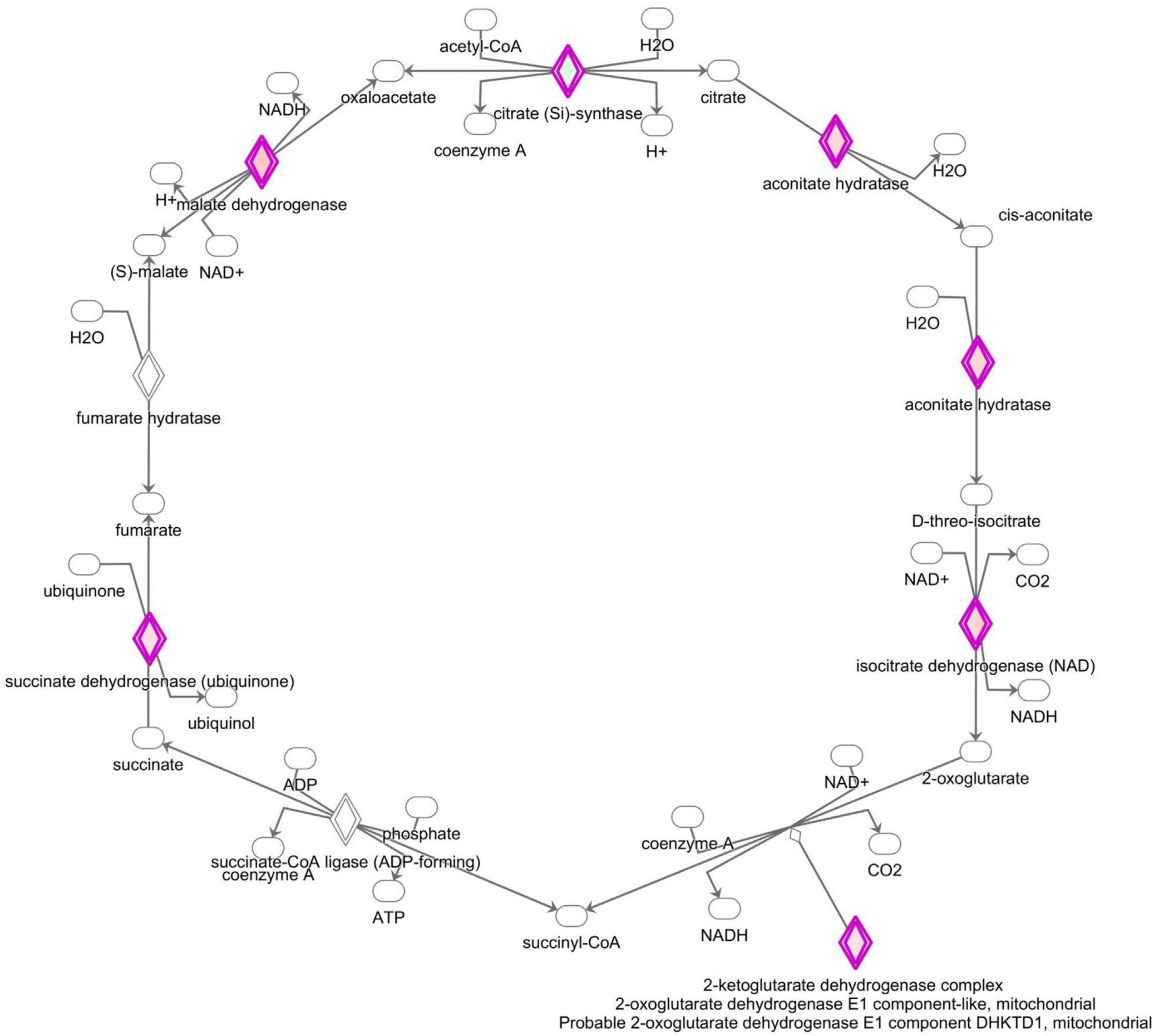


**Supplementary Figure 3: Ingenuity canonical pathway "TCA cycle II" is predicted to be modulated by DE lncRNA XLOC_02093.**

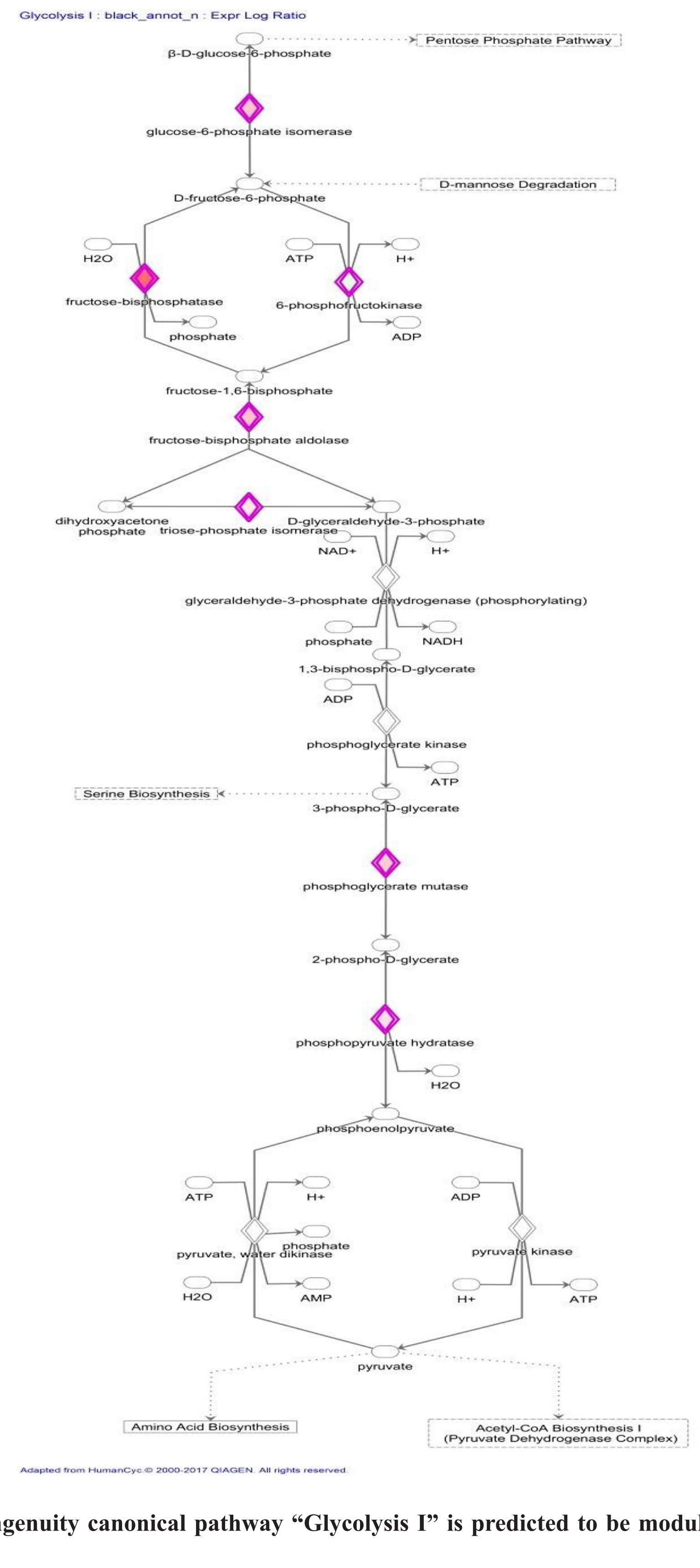


**Supplementary Figure 4: Ingenuity canonical pathway "Glycolysis I" is predicted to be modulated by DE lncRNA XLOC_02093.**

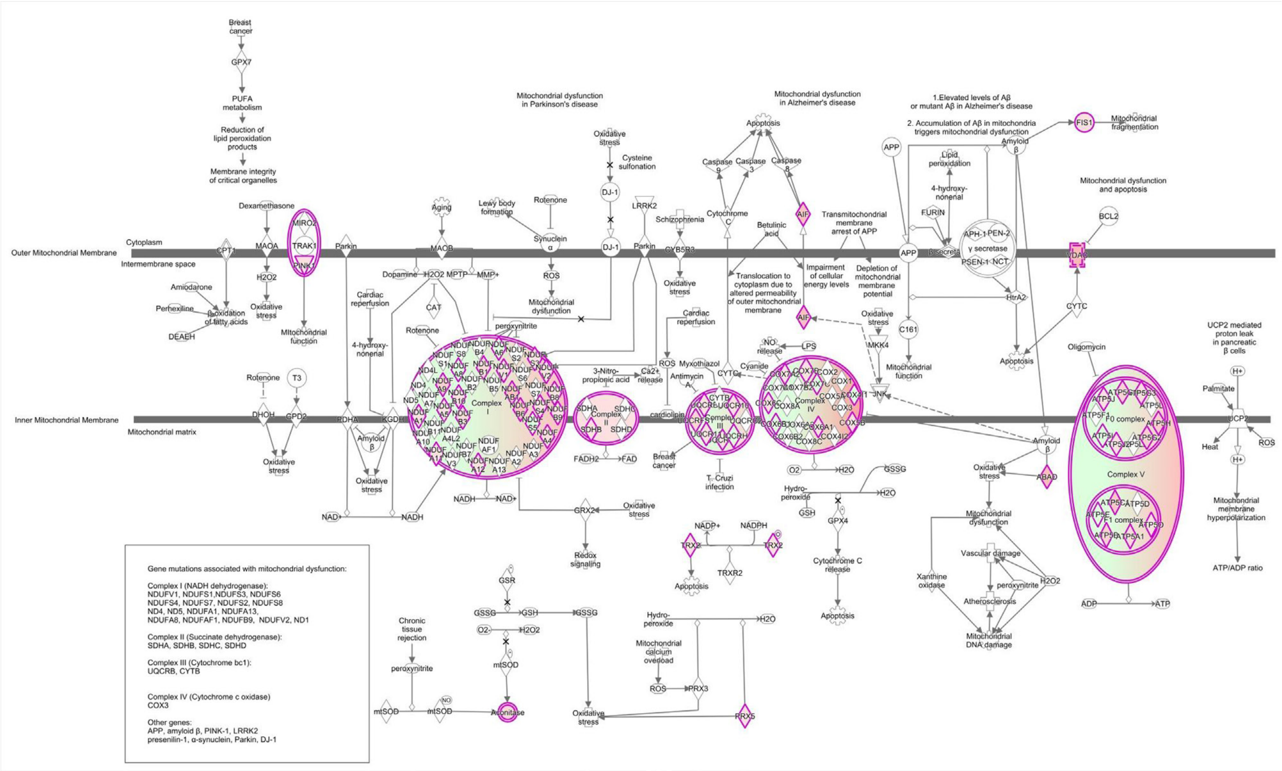


**Supplementary Figure 5: Ingenuity canonical pathway "Mitochondrial dysfunction" is predicted to be modulated by DE lncRNA XLOC_02093.**

**Supplementary Table 1: LncRNAs predicted concordantly by the intersection between the alignment-free lncRNA prediction tools.**

**See Supplementary File 1**

**Supplementary Table 2: Novel mRNAs predicted concordantly by the intersection between the alignment-free novel mRNA prediction tools.**

**See Supplementary File 2**

**Supplementary Table 3: Sequence similarity of lncRNAs predicted concordantly by the alignment-free bioinformatic tools CNCI, PLAR and PLEK with lncRNAs deposited in the NONCODE database.**

**See Supplementary File 3**

**Supplementary Table 4: Tissue-specific expression patterns of DE lncRNAs in other RNA-seq datasets.**

**See Supplementary File 4**
